# The structure of scleractinian coral skeleton analyzed by neutron diffraction and neutron computed tomography

**DOI:** 10.1038/s41598-020-69859-2

**Published:** 2020-07-30

**Authors:** Tatiana I. Ivankina, Sergey E. Kichanov, Octavian G. Duliu, Safa Y. Abdo, Mohamed M. Sherif

**Affiliations:** 1 Frank Laboratory for Neutron Physics, Joint Institute for Nuclear Research, 6, Joliot Curie str, 141980 Dubna, Russian Federation; 2 Department of Structure of Matter, Earth and Atmospheric Physics and Astrophysics, Faculty of Physics, University of Bucharest, 405, Atomistilor str., 077125 Magurele, Ilfov Romania; 3 Faculty of Sciences, Cairo University, Al Orman, Giza Governorate 12613 Egypt

**Keywords:** Zoology, Ocean sciences, Materials science, Physics

## Abstract

Two analytical methods based on the neutrons high penetrability, i.e. neutron diffraction (ND) and neutron computed tomography (NCT) were used to investigate the structure of the aragonitic skeleton of an exemplar/sample of Dipastraea pallida (Dana 1846), a modern hermatypic coral. ND was used to reconstruct the orientation distribution function (ODF) of the crystalline fibrils which compose the coral skeleton. Accordingly, 684 ND spectra were analyzed using the Rietveld method. The result confirmed the aragonite as the sole mineral component of coral skeleton, allowing to recalculate the ODF of aragonite fibrils and to represent it by means of (100), (010) and (001) crystallographic planes pole figures (PF). Experimental PF showed a remarkable similarity with PF recalculated by considering that all aragonite fibrils are oriented either along the growth axis of polyp cups or perpendicular to this direction. This result confirmed the previous observations based on optical microscopy, proving at the same time the availability of ND for such types of investigations. In turn, NCT evidenced the individual polyp cups, their interlocked 3D rigid porous structure as well as a periodic variation of density which could be attributed to a seasonal influence of the marine environment. Different from the classical X-ray computed tomography, the NCT, in view of neutron high cross-section for hydrogen, demonstrated the presence of a small amount of organic matter, otherwise transparent for X- and gamma rays.

## Introduction

**Figure 1:**
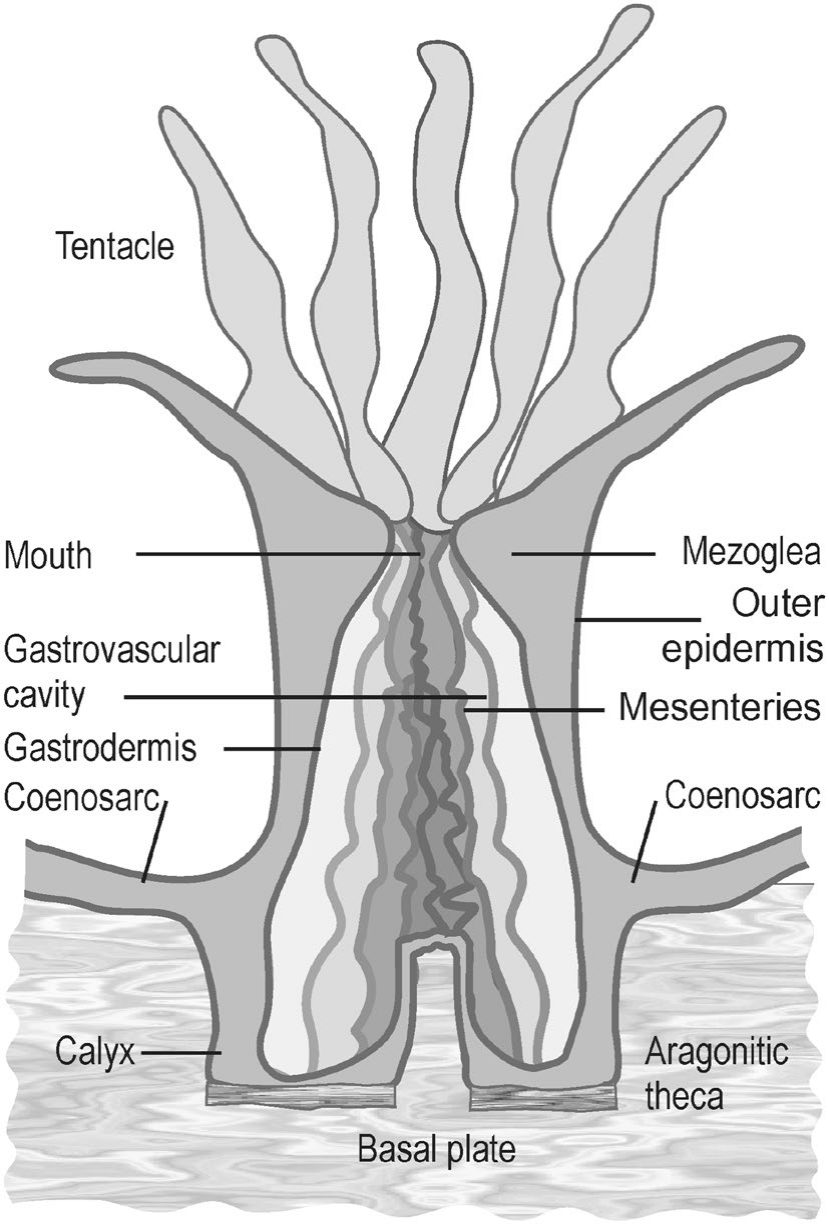
Schematic representation of a polyp. The mesoglea is externally delimited by outer epidermis and by gastrodermis in interior, where it forms an incompletely divided gastrovascular cavity. The polyp is hosted by calyx whose external walls, thecla, are made of aragonite deposited by specialized outer epidermis cell, the calicoblasts. In a colony, all polyps are interconnected by a thin layer of soft tissue, the cenosarc.

Corals are marine invertebrates of significant economic and ecological importance belonging to *Cnidaria* phylum, class *Anthozoa*^1^. They can be solitary or can grow by forming colonies with hundreds of individuals. All corals have the same simple anatomy, basic unit being a sac-like polyp with a radial or radial-bilateral symmetry divided by internal sheet-like radial partition membranous tissue called mesenteries. Coral tissue, consists of a gelatinous substance called mesoglea sandwiched between a layers of inner cells called gastrodermis and an external layer of cell forming the outer epidermis. Gastrodemis incompletely divide the internal (gastrovascular) cavity of polyp in a multiple of six compartments as the case of *Hexacorallia* subclass^2^ or eight as in the case of *Octocorallia* subclass^3^. The polyp is hosted in a protective cup called calyx whose walls, called theca, are made of aragonite fascicles disposed both parallel and perpendicular to polyp growing axis (Fig. 1).

The most representative members of the *Hexacorallia* subclass are stony corals (Order *Scleractinia*) that build themselves a hard exoskeleton consisting of thin spicule of aragonite (orthorhombic CaCO_3_) that linked together form a 3D porous network with a remarkable mechanical rigidity. In this case, the specialized calicoblast cells are responsible for deposition of aragonite outside the polyp. This process takes place in a very thin space between calicoblastic epithelium of outer epidermis and the existing coral skeleton. Here, the hydrogen pumps present on the cell walls pump hydrogen ions (H^+^) out of this space to produce more carbonate (CO_3_^2−^) ions that bond with (Ca^2+^) ions existing in the seawater to produce and deposit solid calcium carbonate (CaCO_3_) for their exo-skeleton^4^. This mechanism makes coral sensitive to ocean acidification as there are more HCO_3_^−^ ions but fewer CO_3_^2−^ ions in acidified seawater which determine corals to spend more energy to pump out H^+^ ions from calcifying space to build skeletons^5^.

With few exceptions, scleractinian corals form colonies consisting of thousands of individuals, all of them interconnected and communicate by a soft tissue—the cenosarc (Fig. 1). Depending on species, the size of such colonies can reach meters in height or diameter^6^.

Although aragonite is a metastable polymorph of calcium carbonate, the exoskeleton of scleractinian corals is made entirely of this mineral^7^ whose deposition is mainly controlled by the Mg^2+^/Ca^2+^ ratio in marine water. As, according to^8^, aragonite is precipitated at a Mg^2+^/Ca^2+^ ratio greater then two and the actual marine water has Mg^2+^/Ca^2+^ ratio of 5–12^9^, this could be a good explanation why the exoskeleton of actual corals entirely consists of aragonite.

The presence of aragonite, as the sole mineral component of scleractinian skeleton, was proved by X-ray diffraction (XRD) and Fourier transform Raman (FTR) spectroscopy, as both methods could discriminate between aragonite and calcite, the two polymorphs of calcium carbonate^10^.

According to^11^, scleractinian coral skeleton grows in two distinct modes, i.e. a vertical extension and a radial development in a plane normal to the vertical one. Conversely, it is expected aragonite fibrils to be orientated by following a simple pattern consisting of two preferred orientations: one along the polyp cup walls and the other normal to the first one assuring cups interconnection.

Regarding XRD, it should be mentioning that X-rays used in modern instrumentation are strongly absorbed in most materials. This restricts the use of XRD to thin samples or to samples whose grains are smaller than 20 μm. Distinct from XRD which allows identification of composing minerals, neutron diffraction (ND)^12^ can be used not only to identify the calcium carbonate polymorphs but, also to evidence the spatial orientation of aragonite crystallites with respect to the whole organism^13^. This permits a better understanding of how the calicoblasts contribute to formation of external skeleton. The difference in the quality of information is due to the capacity of neutrons, particles with no electric charge, to cross objects few cm thick, such as, in our case, a slab of coral colony^14^. It should be remarked that, to be used for diffraction measurements, the neutrons should have an associated wavelength comparable with the distances between the nuclei of investigated objects, i.e. about 0.1–0.3 nm which corresponds to thermal neutrons.

In the case of polycrystalline specimens, such as coral skeleton, the best descriptor of crystalline axis orientation is represented by the orientation distribution function (ODF): *f*(*g*), (*g* ∈ *G*), where *G*-space is a finite space of orientations *g* (*g* being defined by the Euler angles {*α*, *β*, *γ*} ≡ *g*; *α* ≥ 0, *β* ≤ 2π, *c* ≤ *γ* ≤ *π*). In our case, ODF was used to characterize the directionality of aragonite bundles in the complex fiber architecture of scleractinian skeleton^14,15^. Graphically, ODF can be represented by pole figures (PF), i.e. the stereographic projections of the crystalline plane normals ***n*** of considered mineral, in our case, aragonite. PFs should be regarded as a graphical representation of a 3D distribution function of normals ***n*** to crystalline planes of sample^16^. This makes ND an useful tool for investigating the internal architecture of scleractinian skeleton.

Imaging methods, like radiography^17–19^ or X-ray CT^20–22^, have been intensively used in the study of scleractinian skeleton. Their task was mainly to evidence the distribution of hard mineral tissue as well as of the influence of external factors such as temperature, stress, contaminants or bleaching.

Complementary to X-ray imaging, neutron computed tomography (NCT) showed useful in revealing the internal structure of a large category of objects including fossils^23^, geomaterials^24^, works of art^25^. All of them contain high scatter cross sections for thermal neutrons elements such as hydrogen^26^.

Accordingly, we have used neutron time-of-flight diffraction to investigate the spatial distribution of aragonite fibrils and NCT to visualize its internal structure and the distribution of remaining organic matter in scleractinian coral exoskeletons.

## Results

### Texture measurements

**Figure 2:**
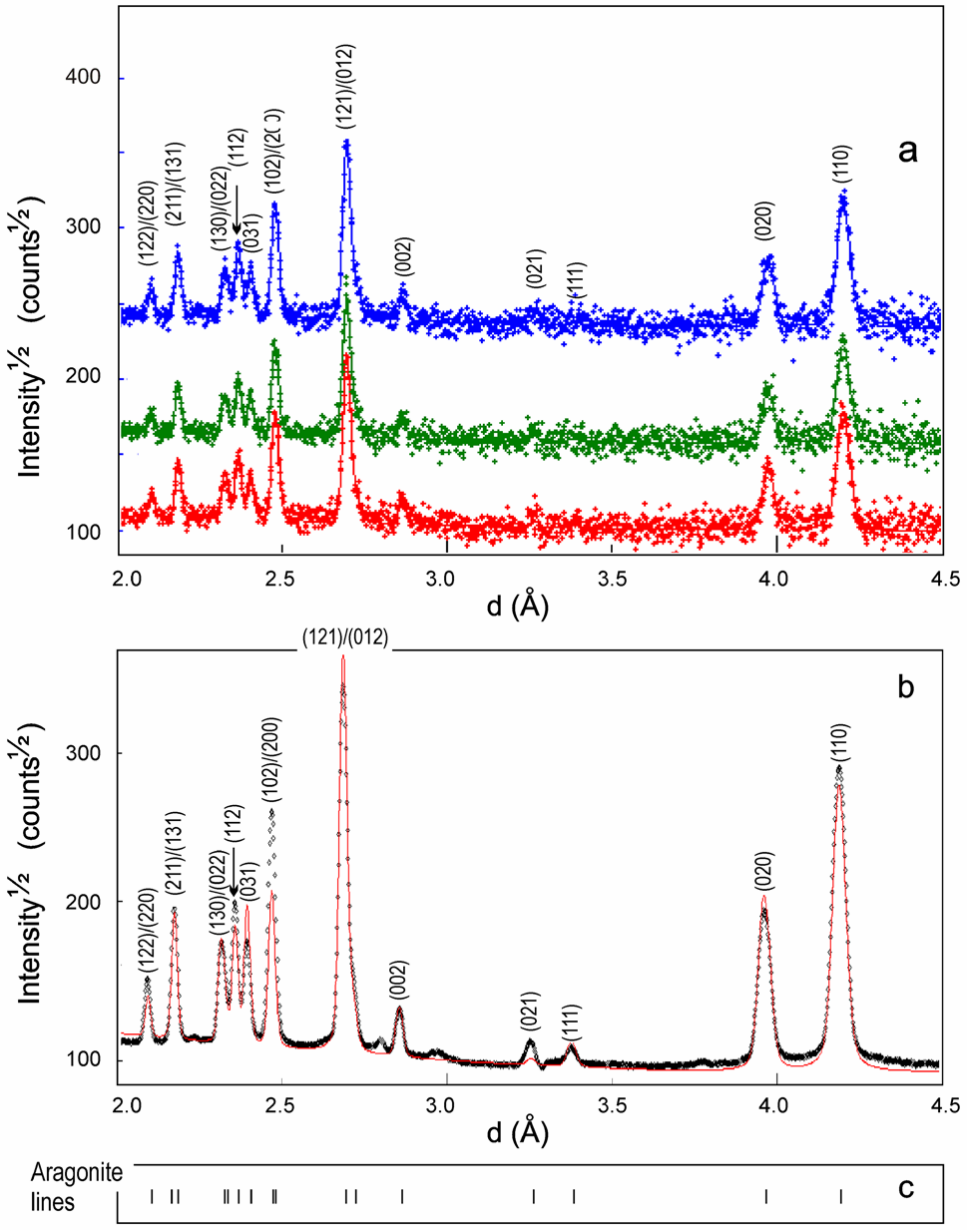
Three neutron diffraction spectra of the coral slab for three different orientation of the sample with respect to thermal neutrons beam (**a**) and the neutron time-of-flight sum diffraction spectrum resulting from the averaging of all 684 spectra (**b**). The experimental points are marked by crosses (**a**) or by circles (**b**) while the corresponding Rietveld fits are represented by continuous lines. On both images, the aragonite Brag’s reflections (**c**) are well evidenced and the presence of other natural carbonates was not confirmed.

The use of TOF diffraction for texture measurements allows for recording of diffraction patterns and measuring several PF simultaneously. For texture measurement, the coral sample was rotated in 10° increments to improve PF coverage. The diffraction data were collected for 60 min for one orientation, which resulted in 36 h per sample, and produced 684 spectra. All diffraction spectra were analyzed using the Rietveld method^27^. Under these conditions, crystallographic texture was better reflected by the diffraction spectra reproduced in Fig. 2a. Here, the relative line intensities which correspond to different orientations of the sample with respect to neutron beam evidence the influence of crystallographic texture on Bragg reflections. By averaging all 684 diffraction spectra we arrived at the spectrum illustrated in Fig. 2b. It consists of 17 lines whose positions are indicated in Fig. 2c and confirmed the presence of aragonite lines only. Moreover, the calculated unit cell parameters: *a* = 4.93187(3) Å, *b* = 7.91945(5) Å and *c* = 5.71342(2) were in perfect agreement with the existing X-ray diffraction (XRD) literature data^28^ for aragonite.

Starting from ND spectra, we have recalculated the ODFs and corresponding PFs of aragonite (100), (010) and (001) crystallographic planes. This was accomplished by using the WIMV algorithm^29^ implemented in the program package BEARTEX^30^. It should be mentioned that WIMV algorithm implies ODF’s calculation based on number of experimental PF. For a quantitative texture analysis (QTA) and in order to minimize the relative error expressed by the residual factor (R factor), the best results were achieved by selecting as input for QTA the experimental PF corresponding to (002), (020) and (021) planes (Fig. 2b).

**Figure 3:**
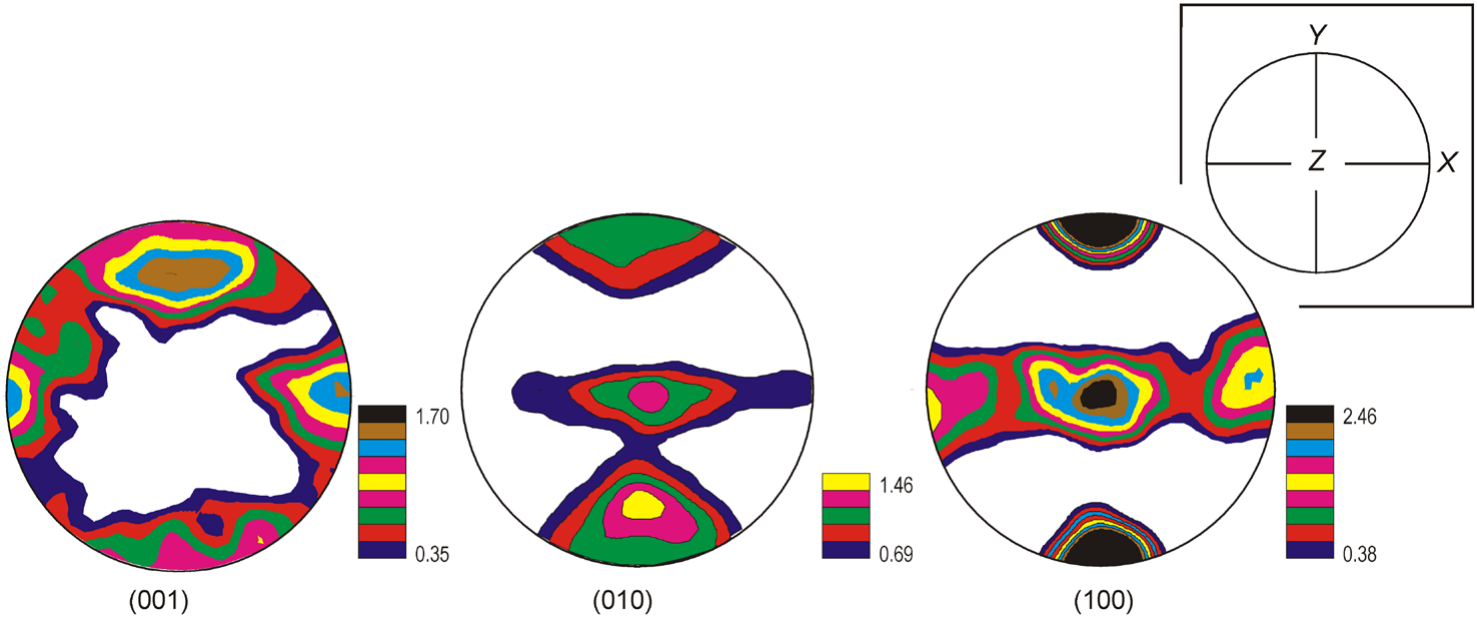
PF recalculated from the neutron diffraction spectrum illustrated in Fig. 2b. The color scale reproduced on the bottom right-hand corner illustrates the isoline intensities expressed in unit of multiples of a random distribution (m.r.d). The inset illustrates the XYZ coordinate system.

PF thus calculated showed some peculiarities attesting a certain degree of organization, far from a random distribution (Fig. 3). Accordingly, PF corresponding to (001) crystalline plane presents four maxima disposed regularly at 90° in the equatorial plane. At the same time, each PF corresponding to (010) and (100) planes show two reciprocal perpendicular maxima, one on the equatorial plane and the other one transversely disposed in the PF center (Fig. 3). Furthermore, it should be remarked as natural coral sample has irregular shape, the PF position with respect to measuring set-up has been slightly shifted. The spatial distribution of fibrils seems to be abnormal which could explain the asymmetry of PF (001) and (010).

### Neutron computed tomography

**Figure 4:**
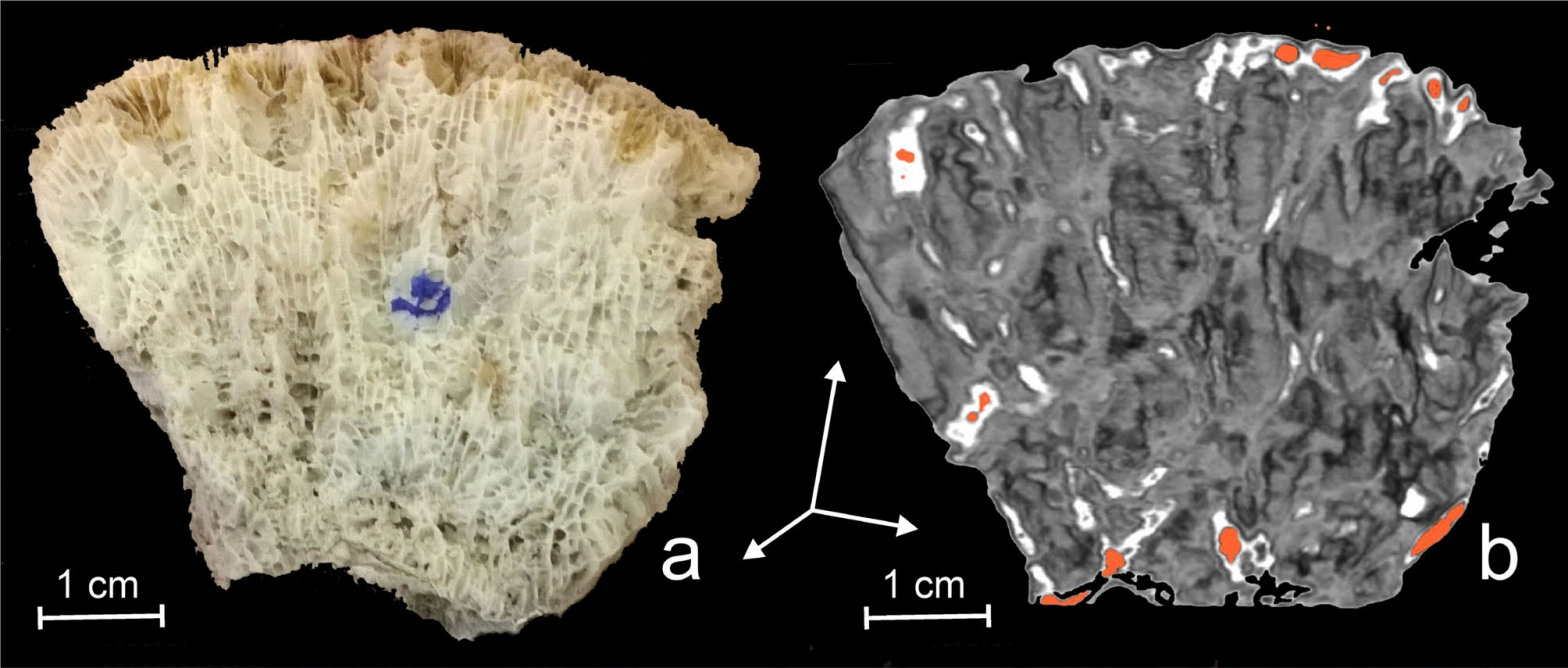
Photo of the investigated *D. pallida* fragment (**a**) and a virtual 3D tomographic model corresponding to coronal slice (**b**). The brownish colour on the superior part photographic image (**a**) corresponds to organic rich residues remained in the skeleton. On tomographic model (**b**) the presence of organic matter, which corresponds to the neutron LCA top 10% highest values, is evidenced in orange color. On both images, the individual calyx walls (theca) appear in oblique sections illustrating consecutive generations of polyps. The lacunar structure of the colony is also well represented in reconstructed model (b).

**Figure 5:**
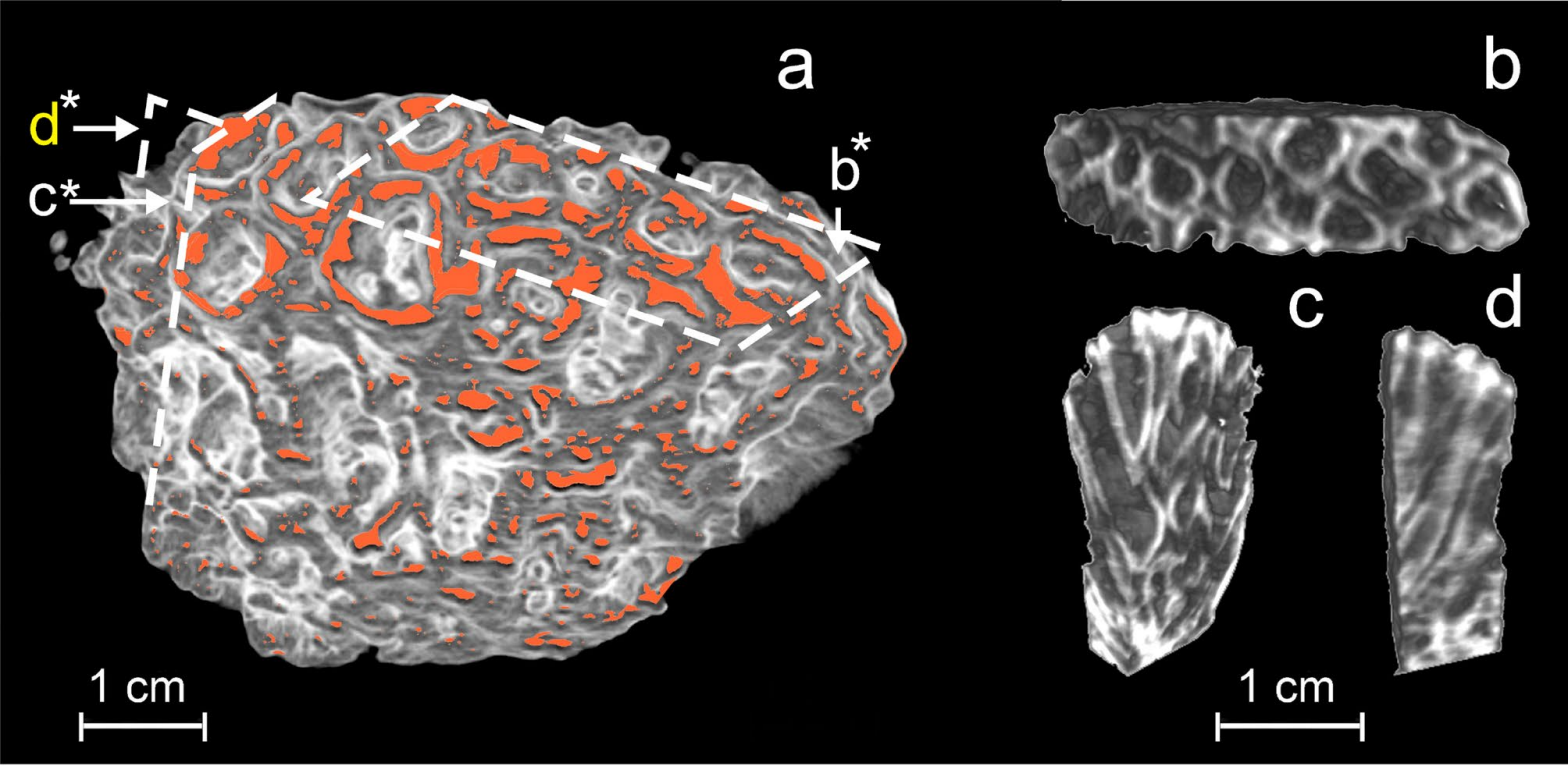
The 3D tomographic models of the superior part of the coral slab (**a**), as well as a transverse (**b**) and two vertical slices (**c**) and (**d**). As in Fig. 4b, the presence of organic rich matter is evidenced in orange color. The tomographic model (**a**) shows with clarity the reciprocal disposition of polyps cups (calyx), also illustrated by the tomographic slices (**b**,**c**). The slices (**c**,**d**) illustrate the vertical disposition of calyx walls (thecla). It should be remarked that on slice (**c**,**d**), the annual growth sections appear in darker hues. On the 3D model (**a**), the positions of (**b**–**d**) slices are marked by asterisk.

The most representative reconstructed tomographic data are reproduced in Figs. 4 and 5. Although the spatial resolution of neutron reconstructed tomographic data is lower than of the photographic ones (Fig. 4a), the tomographic model illustrate more details concerning the 3D internal architecture of coral skeleton. This was achieved by means of differential neutron attenuation (Figs. 4b, 5c,d), which gives better results than optical images.

## Discussion

Concerning the hard tissue of coral skeleton, QTA evidenced the orientation of identically oriented mineral associations, in our case aragonite fibers/bundles with respect to a physical or geometrical coordinate system^31^. Following this model, we were able to analyze the preferred orientations of the principal crystallographic planes of aragonite. This was accomplished within the coordinate system of coral, whose Z axis coincides with the coral vertical axis.

The crystallographic network of aragonite, as reflected by the PF (001), (010) and (100) appeared well-pronounced and symmetrical (Fig. 3). Accordingly, the (001) PFs exhibits two weak reciprocal perpendicular and non-coincident maxima (Fig. 3a). At the same time, the (100) and (010) PFs display two belt like bands perpendicular to the Pfs equator along with a well defined central single maximum perpendicularly to them (Fig. 3b,c).

The degree of crystallographic preferred orientations can be characterized by the texture index, *J*, which represents a bulk measure for the strength of texture:

1 $${J = \frac{{1}}{{{8}\pi^{{2}} }}\sum\limits_{i} {\left[ {f\left( {g_{i} } \right)} \right]^{{2}} g_{i} } }$$

where *g* are the orientations in the G space.

For a random texture, *J* is equal to 1.0. In our case, the texture index *J*, calculated using WIMV algorithm^29^ implemented in the program package BEARTEX^30^, was found to be equal to 1.33; this signifies a weak, less organized, but not a random texture. Consequently, aragonite monocrystals form bundles representing the main constituents of the coral skeleton. Also *J* is a single parameter, in contrast to the ODF, which is a functions of many parameters.

Following this model, texture of aragonite bundles can be easier described using the fibers as individual components whose orientation is distributed along some well defined directions^31–34^.

According to the texture analyzing method utilized in this study, there are two types of components, i.e. “peak” and “fiber” whose orientation is parallel and respectively normal to corallites growth axis^34^. The orientation of peak component is described by three Euler angles (α, β, γ) as well as by the half-width parameter *b* which characterizes the distribution of preferred orientation. “Fiber” component is defined by two unit vectors with parameters θ_y_, φ_y_ and θ_h_, φ_h_ and by the fiber distance *ω*_f_. The first vector is the fiber direction (“skeleton line”) in the sample coordinates, and the second one is the fiber direction in the crystal coordinates (Table 1).

**Table 1:** The main parameters of ideal texture components: α, β, γ are Euler angles, *b* represents the angular distribution half-width, θ_*y*_*, *φ_*y*_ and θ_*h*_*, *φ_*h*_ are the polar coordinates of two vectors which define fiber axis in the sample and crystal coordinate system, *ω*_*f*_ is “fiber distance”.

	α	*β*	γ	θ_*h*_	φ_*h*_	θ_*y,*_	φ_*y*_	*b*	*ω*_*f*_
Peak 1	180°	90°	0°					15°	
Peak 2	180°	80°	90°					15°	
Fiber				90°	90°	0°	0°		30°

For a better understanding of the spatial distribution of aragonite bundles which form coral skeleton, we have fitted the experimental PFs reproduced in Fig. 3 following the Barnes and Luogh^11^ model. Consequently, we have used a linear combination of three texture components: aragonite bundles oriented along the individual corallites axes, in our case Z axis, and two aragonite bundles, normal to the previous ones.

**Figure 6:**
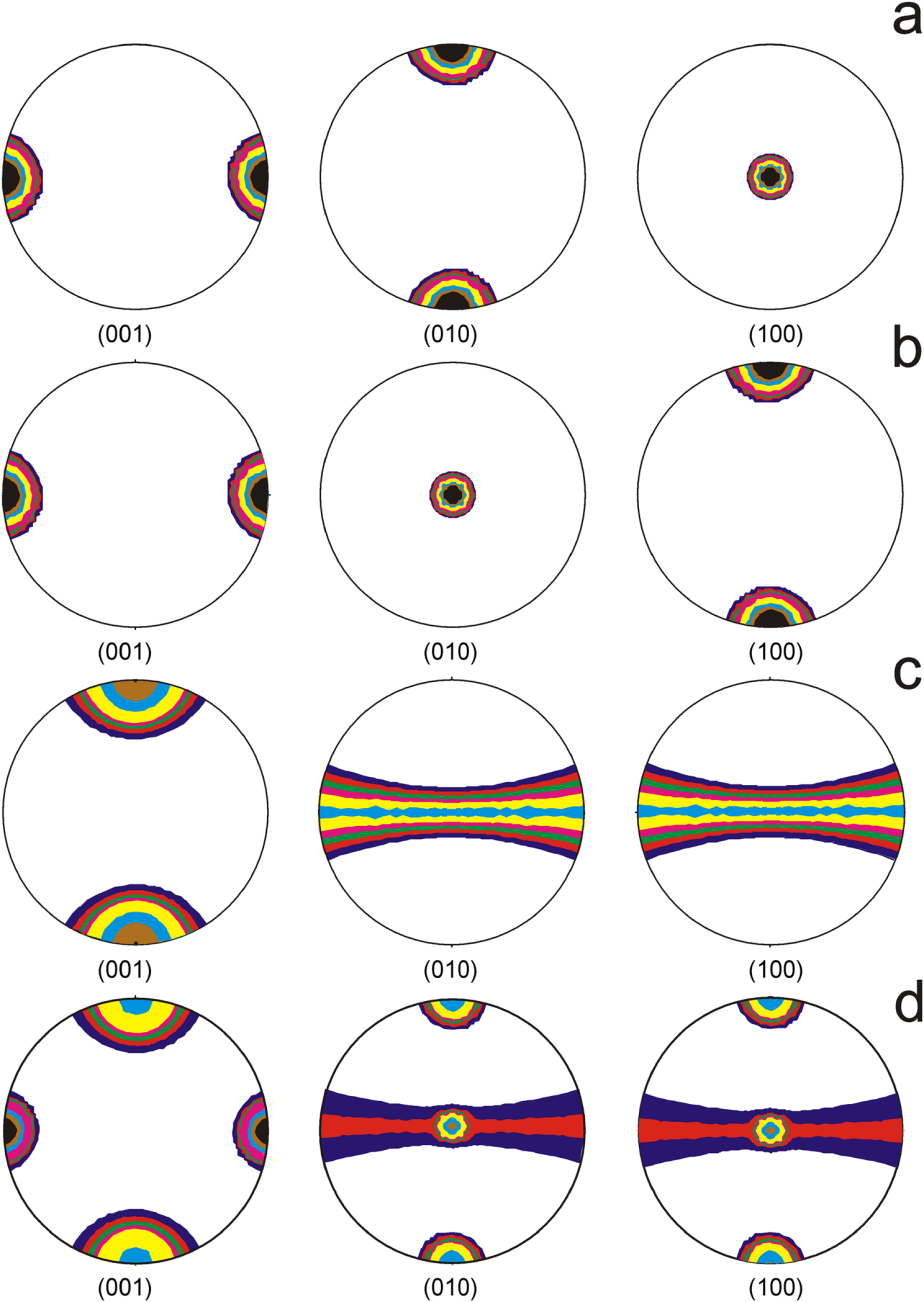
Simulated model of the texture components (fiber and peaks) and their parameters whose combination could explain the principal PFs reproduced in Fig. 3. The PF (**a**–**c**) correspond to different orientations of peaks and fiber parallel to the corallites grow axis while the PF (**d**) represents their superposition showing a remarkable resemblance to experimental PF reproduced in Fig. 3.

While the PF (Fig. 6a,b) correspond to the vertical growth axis of the coral, the PF reproduced in Fig. 6c correspond to the normal to grow axis fibrils which assure the coral skeleton rigidity. The superposition of these three components is reproduced in Fig. 6d. To simulate the natural distribution of aragonite bundles, we have included a certain degree of fluctuation of the Euler angles (*α*, *β*, *γ*) characterized by a *b* parameter of about 30°.

This model does not take into account any randomly oriented fibers. Regardless this approximation, PF illustrated in Fig. 6d appear to be in god correlation with experimental PF reproduced in Fig. 3.

By comparing the experimental PF reproduced in Fig. 3 with the calculated ones illustrated in Fig. 6d, it appears that PF presents the same features. At the same time, PF appear more distorted in the case of experimental ones, most probable due to some imperfection of coral skeleton.

This observation proved that aragonite fibrils, which compose the coral skeleton, are not randomly oriented but follow a well established pattern as Barnes and Luogh^11^ suggested.

As for the coral skeleton structure, a better interpretation of the information furnished by the NCT can be done by comparing the optic (Fig. 4a) and the corresponding 3D volume rendering tomographic model (Fig. 4b). On the photographic image, we observed only the lacunar structure of coral colony skeleton with a multitude of small empty spaces allowing different polyps to communicate. Due to the high resolution of the photographic image, we could observe at the superior extremity of photography (Fig. 4a) the fragments of septa. In contrast, on the tomographic data (Fig. 4b), the internal structure of skeleton offers better clarity. Here, due to dependence of neuron beam attenuation on wall thickness, the distribution of individual coral cups (calices) is more acuratelly illustrated.

Due to the fact that hydrogen has the greatest linear attenuation coefficient (LAC) for neutrons, in order to evidence the presence of organic matter rich in hydrogen, we have selected the voxels with top 10% highest value and represented them in shades of orange (Figs. 4b, 5a).

More details on skeleton morphology are provided in Fig. 5b,c. The reciprocal distribution of calices is well represented on tomographic slice (Fig. 5b). Although the spatial resolution does not allow visualizing internal septa, this image shows with clarity the walls (theca) of individual cups (calices) as well as the way they are interlocked.

The same image helped us to estimate NCT spatial resolution at about 0.15 mm, enough to represent the most important skeleton features. Their presence is evidenced by an alternation of lighter and darker bands (Fig. 6c,d), similar to those previously as seen on thin section radiography of the *Porites* sp.^11^. Such features, in our opinion, could be associated with polyp annual growth, as reported in^35^.

**Figure 7:**
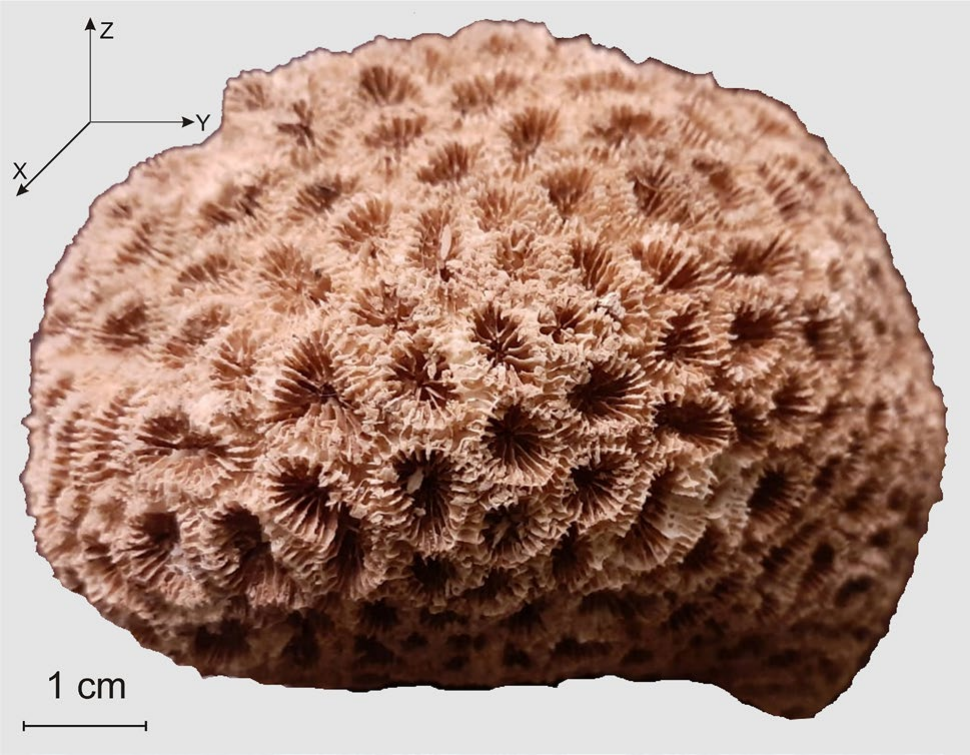
The photographic image of the investigated *Dipastraea pallida* (Dana 1846) coral. The individual cups (calyx) which appear on the coral surface are encircled by a multitude of six septa.

The most intuitive model of the *D. pallida* colony is a beehive like structure which starts from an individual polyp, growths radially by adding consecutive generation which gives the colony an almost hemispheric shape, and whose surface is made of living polyps (Figs. 4a,b, 5a, 7).

Although ND and NCT are based on the same physical principles as X-ray ones, the lack of electric charge and the presence of rest mass, make neutrons quite different from the X-ray as far as, the interaction mechanism is concerned. In the case of *D. pallida*, ND not only to confirm the aragonite as the exclusive mineral component of exoskeleton, but also shows the spatial non-randomly distribution of aragonitic fibrils that compose the skeleton. In contrast to X-ray CT, the NCT 3D representation of coral skeleton shows the spatial distribution of minute amounts of organic matter.

## Methods

### Corals

For our experiments, we have used a fragment of *Dipastraea pallida* (Dana 1846) scleractinian corals (Fig. 7) manually collected at depth between 5 and 10 m southern of Al Saleef port (15.308° S, 42.988° E), Yemen Republic. After being collected, the specimen was washed with seawater, put into plastic bag, transported to University of Cairo laboratory, air dried and shipped to the Joint Institute for Nuclear Research (JINR), Dubna, Russian Federation. By using a water saw, a 6 cm × 6 cm × 1.8 cm slab was prepared for further ND and NCT investigations.

### Texture analysis with neuron diffraction

ND texture investigations were performed using the SKAT time-of-flight (TOF) neutron diffractometer^36^ at the pulsed IBR-2 nuclear reactor of the JINR Frank Laboratory of Neutron. The SKAT TOF diffractometer permits the use of Rietveld analysis (RTA) for texture evaluation^27^.

This procedure allows for a more accurate separation of overlapping PF, in the case of texture investigation of polymineralic rocks, which is not the case of PF inversion^13^.

The SKAT texture diffractometer was equipped with three banks of neutrons detectors arranged around the incident neutron beam at diffraction angles 2θ equal to 65°, 90° and 135°. Each detector bank consisted of 19, 19 and 13 individual single-tube, Gd collimated ^3^He neutron detectors^12,27^. For the current measurements we have used only the detector bank at the scattering angle of 2θ  = 90°.

The sample was placed in the center of the median detector ring, and was rotated by a goniometer whose axis was oriented at 45° with respect to the incident neutrons beam. SKAT diffractometer uses neutron beams with a diameter of 150 mm allowing to measure samples with a volumes up to 150 cm^3^, significantly increasing grain statistics. With this set-up, the samples needed only minimal preparations which simplify the measurements, providing a sufficient number of grains which satisfies the Bragg’s law. A complete description of SKAT texture diffractometer can be found in^13^.

The final diffraction spectrum produced by the Bragg reflections (*hkl*) was obtained by rotating the sample with a constant angular step and recorded for a regular 5° × 10° grid.

### Neutron computed tomography

The NCT experiments were performed at the Neutron Radiography and Tomography Facility of the JINR Frank Laboratory of Neutron Physics. This Facility uses the14^th^ neutron beam-line of the IBR-2 high flux pulsed reactor. The neutron beam has a 5.5 × 10^6^ cm^−2^ s^−1^ fluency density and a 150 mm diameter. The distance between the aperture and the neutron detector was of 10 m with a L/D parameter of 200. Neutron radiographic images have been collected by a 200 mm × 200 mm detector system provided with a 0.2 mm thick RC TRITEC Ltd (Switzerland) ^6^LiF/ZnS(Cu) scintillator screen and a high sensitivity camera with a HAMAMATSU S12101 CCD chip. The scintillation screen was used to convert neutron radiation into visible light.

The imaging data were corrected by the camera dark current and normalizing to the image of the incident neutron beam using the ImageJ software^37,38^. A total number of 360 projections with a step of 0.5° were collected for a full 3D image reconstruction. The tomographic reconstruction was performed by a simultaneous iterative reconstruction technique (SIRT) algorithm of the SYRMEP Tomo Project (STP) software^38–40^. VGStudio MAX 2.2 software (Volume Graphics, Heidelberg, Germany) was used for visualization and analysis of reconstructed 3D data. Although the voxel size were of 52 μm × 52 μm × 52 μm, the two-step neutron detection [primary n, α nuclear reaction, secondary ZnS(Cu) scintilator detector] with the use of large, uncollimated neutron beams reduced the spatial resolution of final tomographic reconstruction to about 150 μm. This is significantly lower then X-ray CT, whose resolution can be better than 30 μm. More details on this tomographic system can be found in^41,42^.
